# Cogan’s sign in a patient with suspected post-COVID-19 vaccine-associated myasthenia gravis

**DOI:** 10.1590/0037-8682-0007-2023

**Published:** 2023-06-02

**Authors:** José Wagner Leonel Tavares-Júnior, Manoel Alves Sobreira-Neto, Pedro Braga-Neto

**Affiliations:** 1 Universidade Federal do Ceará, Divisão de Neurologia, Departamento de Clínica Médica, Fortaleza, CE, Brasil.; 2 Universidade Estadual do Ceará, Centro de Ciências da Saúde, Fortaleza, CE, Brasil.

**Keywords:** COVID-19, SARS-CoV-2 Infection, Myasthenia Gravis

## Abstract

The Cogan’s sign is indicative of myasthenia gravis. This is the first report of neurological signs in a patient with post-COVID-19 vaccine-associated myasthenia gravis in Brazil. In this case, a previously healthy 68-year-old woman presented with proximal limb weakness, left ptosis, and diplopia 1 month after receiving her fourth dose of the COVID-19 vaccine. Neurological examination revealed the presence of Cogan’s sign, and she recovered rapidly after treatment. To our knowledge, this is the first reported case of myasthenia gravis associated with the COVID-19 vaccine in Brazil.

## INTRODUCTION

The Cogan’s sign is characterized by a transient overshoot twitch of lid retraction after a sudden return of the eyes to their normal position following a period of downward gaze. It is caused by a neuromuscular junction disorder known as myasthenia gravis^1^.

Myasthenia gravis is an autoimmune disease characterized by proximal weakness of the limbs and ocular muscles, with an annual incidence of 30 cases per million adults^2^ ^,^ ^3^. Characteristic findings, like autoantibodies against the acetylcholine receptor or a decremental pattern on electroneuromyography with repetitive stimulation, can aid in its diagnosis^4^. 

Autoimmune diseases can occur after vaccination^5^. Vaccines against influenza and hepatitis B have been linked to myasthenia gravis, and the underlying mechanism may involve molecular mimicry^6^ ^-^ ^8^. To the best of our knowledge, this is the first reported case of COVID-19 vaccine-associated myasthenia gravis and the first report of neurological signs in a patient with post-COVID-19 vaccine-associated myasthenia gravis in Brazil.

## CASE REPORT

A search strategy was developed to conduct endless searches in MEDLINE via PubMed. The following descriptors (bold), synonyms, natural language, and Boolean operators were used to cross-check the databases: MEDLINE (medical subject headings [MeSH]: search strategy-(myasthenia gravis) and (COVID-19 vaccine) and (Oxford AstraZeneca). The search was conducted on January 12, 2023.

A previously healthy 68-year-old female patient presented with proximal limb weakness, left ptosis, and diplopia 1 month after receiving her fourth dose of the COVID-19 vaccine, (Oxford-Astra Zeneca®). She consulted a neurologist who requested electroneuromyography with repetitive stimulation of the four limbs, which revealed a decrement pattern, consistent with myasthenia gravis. The absence of clinical symptoms suggestive of infection (such as fever and dyspnea) and laboratory and imaging tests (such as chest radiography, polymerase chain reaction test for COVID-19, leukocyte count, and C-reactive protein test) ruled out infections as possible triggers. Neurological examination revealed the Cogan’s sign (Supplementary material - VIDEO 1 and VIDEO 2). The patient was administered pyridostigmine 60 mg four times daily. However, her condition continued to deteriorate, necessitating admission to the intensive care unit 2 months after the initial diagnosis due to dysphagia, dysarthria, and dyspnea. She was intubated and underwent five sessions of plasmapheresis. Rapid improvement was observed with prednisone (1 mg/kg) and azathioprine/pyridostigmine combination. The patient also underwent thoracic computed tomography, which showed no thymoma, and an anti-acetylcholine receptor antibody test, which showed a positive result. No analysis of the cerebrospinal fluid was performed. She is currently stable neurologically and receives azathioprine 50 mg and prednisone 10 mg daily with pyridostigmine 60 mg six times daily. The patient provided informed consent for the publication of this report.

## DISCUSSION

This case report demonstrated neurological signs in a patient with post-COVID-19 vaccine-associated myasthenia gravis. To our knowledge, this is the first report of post-COVID-19 vaccine-associated myasthenia gravis in Brazil.

The Cogan’s sign, a characteristic of myasthenia gravis, was first described in 1965. It is elicited by instructing a patient to maintain a downward gaze for 15 s, followed by an upward gaze. When positive, the upper lid produces an overshoot^9^.

Other authors have previously described post-COVID-19 vaccine-associated myasthenia gravis, and we found 14 cases in the literature^6^ ^,^ ^10^. Ramdas et al. reported seven new cases and reviewed seven other cases in the literature. Their study included individuals aged 13 to 83 years, with symptoms developing 2 to 14 days after vaccination^6^. 

Myasthenia gravis cases after COVID-19 infection and COVID-19 vaccination may be caused by immune dysregulation^5^ ^,^ ^11^. A few cases of post-COVID-19 myasthenia gravis that may have been caused by molecular mimicry have been described^12^. In addition, post-COVID-19 vaccine-associated myasthenia gravis may occur because of a potential bystander effect from autoreactive T-cell activation and activation of the toll-like receptor pathway^7^ ^,^ ^8^.

The limitations of our study include the fact that it involved a single case, and the period between vaccination and the onset of symptoms was longer than that described in previously reported cases. It is important to note that there are several types of COVID-19 vaccines, and this case demonstrates a rare adverse reaction to only one type. To the best of our knowledge, this study is the first case of post-COVID-19 vaccine-associated myasthenia gravis in Brazil, and the neurological signs were documented on video. This case showed neurological signs in a patient in an atypical context, which will assist medical students and doctors in considering myasthenia gravis after COVID-19 vaccination.

## SUPPLEMENTARY MATERIAL

**VIDEO 1** (https://youtu.be/JKitm9dxSYM)

## Appendix

**VIDEO 2** (https://youtu.be/Dmwrvx7Ccq0)
